# Supplementary figures and images for: Circulating blood extracellular vesicles as a tool to assess endothelial injury and chemotherapy toxicity in adjuvant cancer patients
Source: PLoS One. 2020 Oct 27;15(10):e0240994. doi: 10.1371/journal.pone.0240994 (PMC7591065; doi:10.1371/journal.pone.0240994)

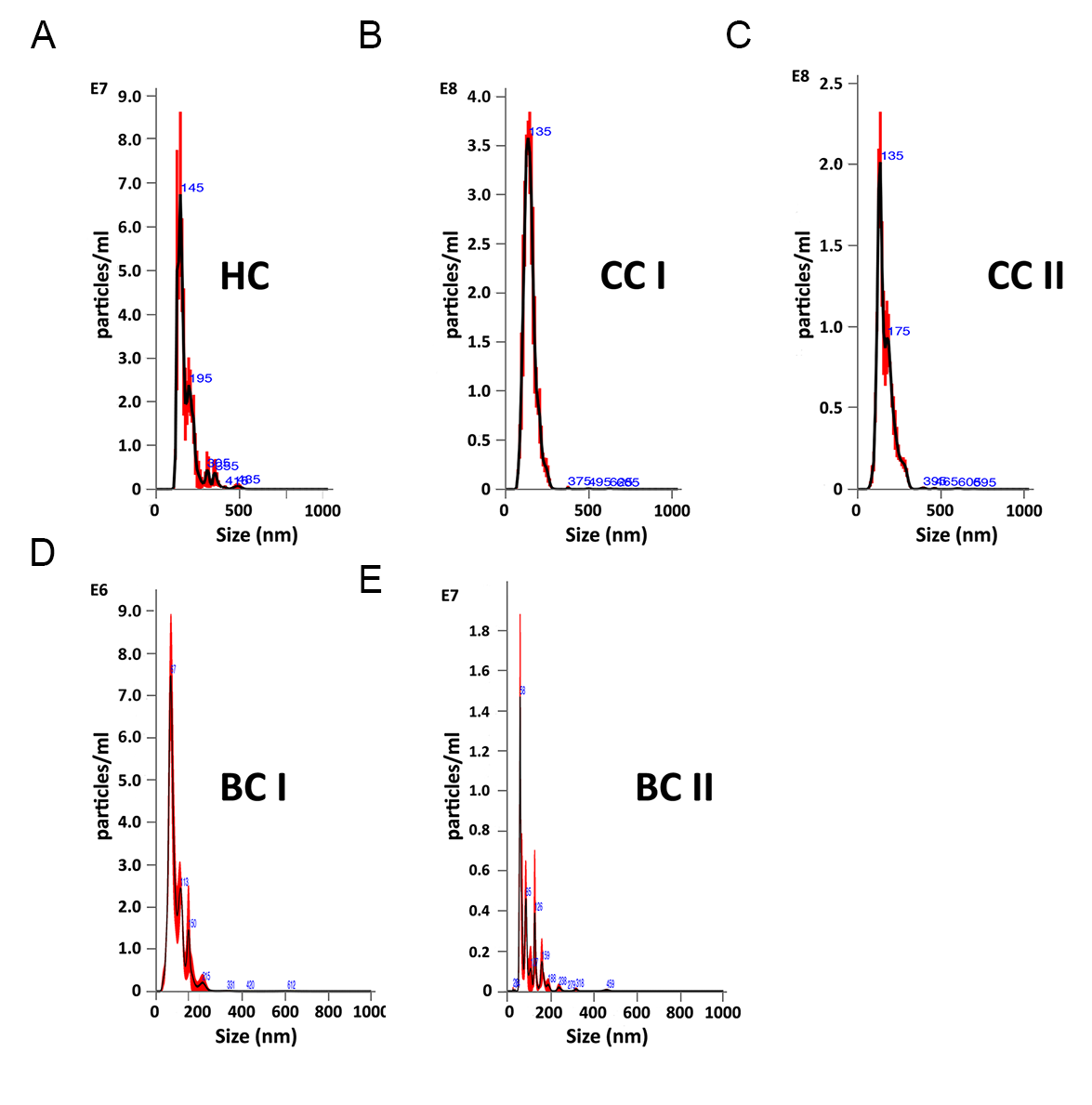

Supplement: S1 Fig — The graphs summarized 3–4 repeats of analysis for each sample and expressed as Averaged FTLA Concentration / Size for HC-EVs (A), CC I-EVs (B), CC II-EVs (C), BC I-EVs (D), and BC II-EVs (E). (TIF) [file pone.0240994.s001.tif]

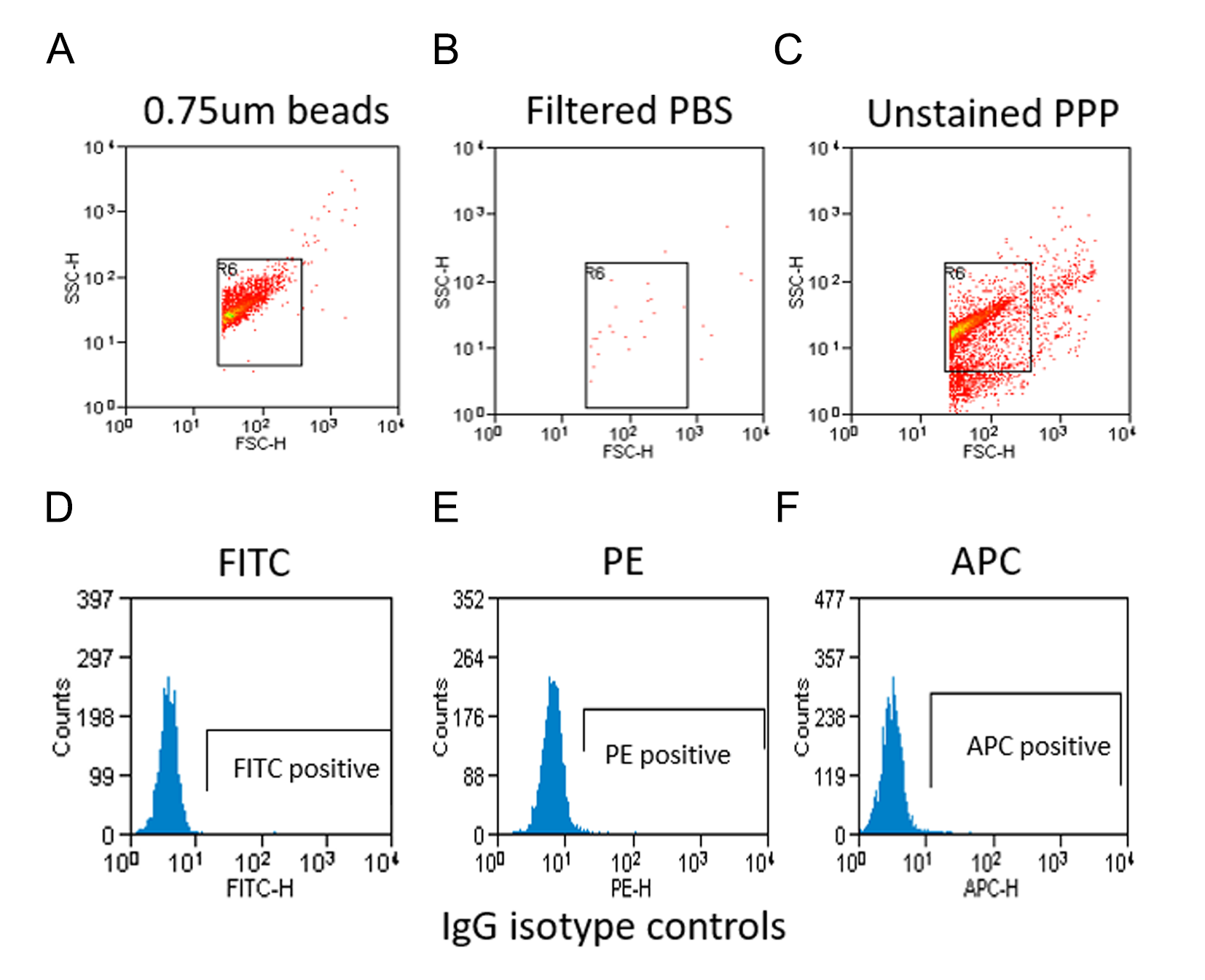

Supplement: S2 Fig — (A) Estimate gate for 0.75 um beads (r1) used for EVs size evaluation. (B) Filtered (0.22um) PBS. (C) Unstained sample PPP-EVs distribution. Forward Scatter (FSC) information about particle size vs. Side Scatter (SSC) information about particles granularity. EVs samples were labeled with IgG isotype controls; the graph presented the fluorescent intensity. The gate was set for positive label area, (D) IgG isotype controls FITC (E) IgG isotype controls PE, and (F) IgG isotype controls APC. (TIF) [file pone.0240994.s002.tif]

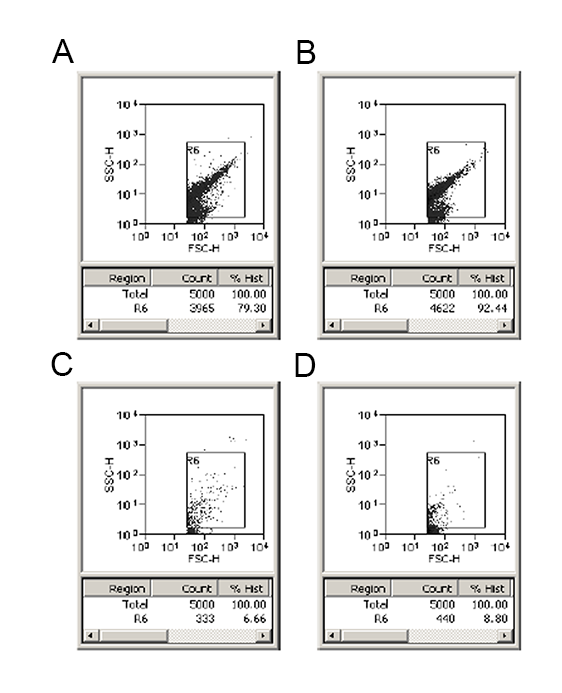

Supplement: S3 Fig — Unstained PPP EVs are located in the same area were 0.75μm beads are located (R6, S1A Fig). (A). HC11–79% EVs located at R6 (B). BC2 II: 92% EVs located at R6. Treatment with 1% triton-x100 reduced EVs number in the R6 area by above ten times. (C). HC 11: EVs located at R6, decreased to 6.6%. (D). BC2-II: EVs, located at R6, reduced to 8.8%. (TIF) [file pone.0240994.s003.tif]
