# Supplementary material for: Circulating blood extracellular vesicles as a tool to assess endothelial injury and chemotherapy toxicity in adjuvant cancer patients
Source: PLoS One. 2020 Oct 27;15(10):e0240994. doi: 10.1371/journal.pone.0240994 (PMC7591065; doi:10.1371/journal.pone.0240994)
Supplement: S1 Table — (PDF) [file pone.0240994.s004.pdf]

S1 Table. EVs membrane antigens characteristics and parameters.

EVs in poor platelet plasma were isolated from blood samples of patients and HC by a series of centrifugations. EVs were labeled with fluorescent antibodies for membrane antigens characterized: endothelial cells (EC), white blood cells, coagulation proteins, and growth factor receptors.

**EVs markers**<br>**Mean, Median, quarrel range**	**HC-BC**	**BC I**	**BCII**	**HC-CC**	**CCI**	**CC II**	**One-way ANOVA,**<br>**Bonferroni's Multiple Comparison Test,**<br>**P-value**
**Cancer markers:**<br>MUC1 (CD227)	34.42<br>39.50<br>26.85-<br>41.40	44.88<br>50.25<br>24.85-<br>60.30	53.30<br>36.90<br>17.15-<br>60.20	27.73<br>28.40<br>18.10-<br>41.00	24.18<br>15.55<br>7.650-<br>41.55	29.63<br>24.30<br>11.20-<br>35.00	
EPCAM	3.295<br>3.800<br>0.400-<br>6.050	5.623<br>1.820<br>0.645-<br>8.500	7.647<br>1.080<br>0.370-<br>7.675				
A33				6.162<br>3.400<br>1.850-<br>11.15	18.31<br>8.050<br>3.800-<br>39.65	17.61<br>12.20<br>5.250-<br>27.00	
**RBC:** (CD253)	47.05<br>51.30<br>40.70-<br>59.60	40.23<br>37.35<br>17.10-<br>64.70	40.79<br>32.00<br>14.10-<br>65.60	35.07<br>26.85<br>11.45-<br>52.45	53.48<br>57.10<br>43.45-<br>64.30	50.02<br>49.30<br>38.40-<br>64.80	
**Platelet:** (CD41)	32.11<br>23.50<br>6.700-<br>50.55	16.12<br>11.89<br>2.765-<br>26.71	15.70<br>8.400<br>2.300-<br>24.00	16.78<br>11.20<br>4.800-<br>23.40	6.855<br>4.650<br>1.050-<br>11.75	11.34<br>8.850<br>4.450-<br>14.80	HC for BC vs. BC I and vs. BCII, $P < 0.05$

**Activated platelet**: (CD62P)	4.485<br>2.080<br>0.850-<br>5.350	4.353<br>1.430<br>0.505-<br>8.450	6.022<br>3.000<br>0.600-<br>9.300	4.373<br>4.400<br>0.0-<br>5.400	2.360<br>0.700<br>0<br>0.0-<br>2.150	3.090<br>1.150<br>0.0-<br>4.100	
**Endothelial:** CD144	23.46<br>25.00<br>5.550-<br>33.60	47.73<br>47.50<br>10.29-<br>79.30	56.13<br>58.00<br>31.30-<br>84.55	33.28<br>29.30<br>23.10-<br>47.75	48.79<br>52.40<br>32.20-<br>61.40	50.33<br>52.00<br>32.00-<br>63.20	HC for BC vs BC II, P < 0.01<br>HC for CC vs CCII, P < 0.05
CD62E	9.520<br>7.600<br>3.750-<br>9.520	37.43<br>29.92<br>6.20-<br>37.43	32.85<br>21.80<br>4.700-<br>32.85	10.76<br>8.800<br>3.600-<br>10.76	9.786<br>8.400<br>1.700-<br>9.786	8.111<br>8.000<br>1.100-<br>10.60	HC for BC vs:<br>BC I, P < 0.01, BC II, P < 0.05<br>BC I vs CC I, P < 0.001<br>BC II vs CCII, P < 0.01
CD31+41-	3.628<br>2.930<br>1.260-<br>5.200	12.11<br>5.300<br>2.300-<br>21.60	8.473<br>3.600<br>0.5900-<br>15.00	4.331<br>3.400<br>2.150-<br>6.600	10.78<br>6.100<br>3.800-<br>14.20	12.77<br>13.45<br>6.000-<br>18.50	HC for CC vs CCII, P < 0.05
**White blood cells**<br>Leucocyte: (CD11)	4.681<br>3.250<br>1.550-<br>7.800	19.54<br>17.95<br>11.65-<br>28.20	14.56<br>9.750<br>4.700-<br>14.35	7.408<br>4.100<br>1.850-<br>9.00	4.526<br>3.100<br>0.0-<br>7.200	7.663<br>6.900<br>1.300-<br>8.300	HC for BC vs:<br>BC I, P < 0.001; BC II, P < 0.001<br>BC I vs CC I, P < 0.001
Monocyte: (CD14)	12.36<br>12.60<br>5.900-<br>17.60	10.41<br>7.925<br>2.985-<br>13.41	8.835<br>5.175<br>1.530-<br>16.70	9.069<br>9.800<br>3.200-<br>14.60	4.013<br>1.700<br>0.0-<br>5.350	7.853<br>4.700<br>1.950-<br>9.650	BC I vs CC I, P < 0.05
	7.950	1.850	1.810	4.400	0.650<br>0	1.950	
**Coagulation :**	17.98	12.82	16.03	13.96	6.238	6.153	

Annexin V binding to negatively charged phospholipid	12.50	6.065	8.800	9.700	4.600	4.700	
TF	6.595<br>7.100<br>4.20-8.470	19.95<br>12.92<br>6.765-34.59	17.17<br>11.49<br>3.090-30.10	6.500<br>7.100<br>3.20-9.100	18.60<br>19.35<br>10.70-25.95	22.512<br>0.55<br>10.20-30.45	HC for BC vs BC I, P < 0.05;<br>HC for CC vs CC I P < 0.001, HC for CC vs CC II P < 0.01
TFPI	10.93<br>7.70<br>2.950-16.00	10.88<br>8.10<br>2.95<br>17.66	6.347<br>2.680<br>1.800-10.63	6.710<br>5.750<br>2.020-7.850	5.629<br>4.60<br>2.050-8.650	4.731<br>3.550<br>1.100-8.050	
TF/TFPI<br>% Above max value of relative HC	1.670<br>1.423<br>0.497-2.678	6.721<br>2.600<br>0.800-5.100<br>32%	7.223<br>2.220<br>1.130-9.250<br>32%	3.613<br>1.415<br>0.560-7.130	7.573<br>2.865<br>1.315-13.25<br>24%	9.066<br>3.960<br>1.460-8.750<br>19%	
EPCR	3.722<br>3.000<br>1.850-5.700	18.02<br>18.38<br>7.055-25.18	14.17<br>8.525<br>2.850-14.43	3.764<br>2.750<br>1.450-6.450	15.01<br>8.850<br>2.900-20.70	13.37<br>10.95<br>4.900-20.35	HC for BC vs BC I, P < 0.05<br>HC for CC vs CC I, P < 0.05
**Growth factor receptors:**<br>VEGF R1(FLT1)	15.12<br>14.90<br>0.700-28.00	52.41<br>47.41<br>16.45-91.48	48.67<br>37.00<br>11.25-88.53	16.74<br>13.00<br>3.300-30.35	45.90<br>44.90<br>31.05-57.85	45.09<br>44.60<br>26.90-63.30	HC FOR BC VS: BC I, P < 0.001<br>BCII, P < 0.01<br>HC for CC vs: CC I, P < 0.05<br>CCII, P < 0.05

VEGF R2(KDR)	11.01<br>7.350<br>3.850-<br>14.00	37.94<br>39.90<br>15.32-<br>56.66	36.48<br>33.31<br>22.58-<br>42.97	6.029<br>5.000<br>4.000-<br>9.700	26.93<br>30.70<br>19.75-<br>33.40	20.32<br>13.50<br>6.850-<br>30.85	HC FOR BC VS: BC I, P < 0.01<br>BCII, P < 0.01<br>HC for CC vs CC I, P < 0.01
