# Supplementary material for: Circulating blood extracellular vesicles as a tool to assess endothelial injury and chemotherapy toxicity in adjuvant cancer patients
Source: PLoS One. 2020 Oct 27;15(10):e0240994. doi: 10.1371/journal.pone.0240994 (PMC7591065; doi:10.1371/journal.pone.0240994)
Supplement: Attachment — Submitted filename: Response to Reviewers.docx [file pone.0240994.s005.docx]

June 30, 2020

To the Editor of **PLOS ONE**

Attached is our revised version of the manuscript  **"**Circulating Blood Extracellular Vesicles as a Tool to Assess Endothelial Injury and Chemotherapy Toxicity in Adjuvant Cancer Patients" for consideration in your journal.

After incorporating and implementing all the necessary corrections and adjustments suggested by the reviewers, we now believe that our work is suitable and ready for publication in the journal.

Specifically, we addressed the following issues:

1. We sent the manuscript to professional editing as required by the journal style.
2. We added the necessary details and information in the Ethics statement and the Methods sections regarding participant consent for the colon cancer population group.
3. We now included additional information about the participant recruitment method and the demographic details of our participants in two new tables (**see Table-1 and Table-2**). A description of where participants were recruited and where the research took place, including inclusion criteria, are now clearly indicated in the method (**Study details and ethical approvals**) and results (**Study cohort**) sections.
4. We provided the necessary text regarding the research study sample size in the Methods (**see Statistical analysis**).
5. As suggested by Reviewer-2, the term toxicity was omitted, and the Abstract sentence was rephrased accordingly.
6. The 2nd sentence of the Introduction was corrected and rephrased, as suggested by reviewer 2.
7. As suggested by reviewer 2, the discussion of patient demographics and tumor characteristics is now in the results section.
8. As suggested by reviewer 2, the results section now starts with the description of patient clinical demographics.
9. As for justification of the choice of fractionation method, and a full description of the fractionation protocol including information about starting and final volumes, concentration factor, and the centrifuge, rotor, and any braking, etc. as requested by reviewer 1:

This study does not intend to separate between the vesicles sub-populations such as Microvesicles (MVs ) and exosomes. The tests performed, as well as the results and conclusions, refer to the entire population of vesicles measuring between 30nm and approx. 500nm in PPP.

Results of a worldwide survey on the techniques used for the isolation and characterization of extracellular vesicles (Gardiner C. J Extracell Vesicles. 2016) show that differential centrifugations are most widely used as the primary isolation method. Notably, increasing centrifugal strength is used for pellet cells (300g), microvesicles (10,000g), and exosomes (100,000g) (Thery C, Curr Protoc Cell Biol. 2006. Chapter 3:Unit 3). Nanoparticle tracking analysis (NTA) has confirmed the mean size above 200 nm for the pellet of 10,000 g and below 200 nm for the 100,000 g pellet (Kowal J. Proc Natl Acad Sci U S A. 2016). However, differential centrifugations cannot provide "pure separation" between exosomes and MVs. For example, Kowal J.et al (PNAS 2016) used EV pellets, obtained by several centrifugations (3,000g, 10,000g, and 100,000g), all of which contained a mixture of small vesicles <100 nm and large EVs in different proportions. Additional research highlights that, "a sharp separation between EV populations based on size was not possible using sequential centrifugation due to overlapping sizes (Ender F. Int J Mol Sci. 2019). In line with these papers, we choose to characterize the total EVs in PPP, which reflects the physiology and pathology state.

**Clarifications regarding the EV isolation protocol have now been added to the Methods section.**

" Blood samples were collected in tubes containing sodium citrate (3.2%) and EDTA; the time delay between blood collection and plasma preparation was less than 1 hour. Differential centrifugations were done according to the current gold standard for EV isolation (23). Specifically, platelet-poor plasma (PPP) was obtained after two sequential centrifugations (15 min 1500g, 240C) within an hour of collection and frozen at −80°C. EVs size, concentration, and membrane antigens levels were validated on PPP samples. In addition, EVs pellets were isolated from thawed PPP by centrifugation (centrifuge Hettich MIKRO 220R, rotor 1189A; Centrifugation condition: 1 hour, 20,000g, 40C, braking - 0). Liquid supernatants were discarded, and EV pellets were used for additional analysis by western blot (WB) and protein array."

10. For the request to present representative histograms showing the size distributions, and relevant details about the measurement, including the length of the tracking videos, the analysis algorithm used to analyze the data, and variance in their triplicate technical replicates and how did we evaluate the fraction of non-EV particle in their sample.

As we mentioned above, the evaluation was done on total EVs of PPP without fractionation. **Representative histograms showing the size distributions of each patient's subgroup were now added as Supplementary Figure-1**. **The following sentence including the relevant details about the measurement, the length of the tracking videos, the analysis algorithm used to analyze data and variance in their triplicate technical replicates, is now added to the method section**:

" Samples measurements were performed with a NanoSight (NTA) version: 3.1 Software Version build 3.1.54. Temper Samples measurements ature 250C, Viscosity/cP-0.868494, Camera Type –sCMOS, Software settings for analysis were kept constant for all measurements. All samples were diluted in 0.025μm filtered PBS to an appropriate concentration before analysis. At least three 30s videos were recorded per sample in light scatter."

11. The requested information regarding FACS setting and controls are now added to the Methods section under "Evaluating EVs membrane antigens," together with relevant graphs as **Supplement Figure-2**.
12. As for providing a better explanation or excuse for the use of the angiostatin carried inside EVs as a useful potential biomarker as requested by Reviewer-1:

For CC patients and HC, protein array was performed on patients EVs pellets and also on supernatant(S) that discarded after centrifuging of 20,000g 1h. We added new data in (**Figure-6D**) presenting Angiostatin content in EVs fraction and the supernatant fraction after EVs depletion. The figure shows that the majority of Angistatin is located in EVS fraction, and its levels are significantly higher in patients samples compared to HC.

The following sentence was added to the results section " Comparing between proteins content in the EVs and the supernatant fraction demonstrated that the majority of Angistatin is located in EVs fraction and its levels significantly higher in patients samples compared to HC (**Figure-6D**)".

We thank the reviewers for their constructive comments and suggestions, improving the manuscript clarity and understanding. We are now enclosing the revised manuscript with all necessary changes indicated, hoping it now stands with the high standards of publication in PLOS-ONE

Sincerely yours

Gil Bar-Sela MD
